# A Temporal Dimension to the Influence of Pollen Rewards on Bee Behaviour and Fecundity in *Aloe tenuior*

**DOI:** 10.1371/journal.pone.0094908

**Published:** 2014-04-22

**Authors:** Karl J. Duffy, Steven D. Johnson, Craig I. Peter

**Affiliations:** 1 School of Life Sciences, University of KwaZulu-Natal, Scottsville, Pietermaritzburg, South Africa; 2 Department of Botany, Rhodes University, Grahamstown, South Africa; Royal Holloway University of London, United Kingdom

## Abstract

The net effect of pollen production on fecundity in plants can range from negative – when self-pollen interferes with fecundity due to incompatibility mechanisms, to positive – when pollen availability is associated with increased pollinator visitation and fecundity due to its utilization as a reward. We investigated the responses of bees to pollen and nectar rewards, and the effects of these rewards on pollen deposition and fecundity in the hermaphroditic succulent shrub *Aloe tenuior*. Self-pollinated plants failed to set fruit, but their ovules were regularly penetrated by self-pollen tubes, which uniformly failed to develop into seeds as expected from ovarian self-incompatibility (or strong early inbreeding depression). Bees consistently foraged for pollen during the morning and early afternoon, but switched to nectar in the late afternoon. As a consequence of this differential foraging, we were able to test the relative contribution to fecundity of pollen- versus nectar-collecting flower visitors. We exposed emasculated and intact flowers in either the morning or late afternoon to foraging bees and showed that emasculation reduced pollen deposition by insects in the morning, but had little effect in the afternoon. Despite the potential for self-pollination to result in ovule discounting due to late-acting self-sterility, fecundity was severely reduced in artificially emasculated plants. Although there were temporal fluctuations in reward preference, most bee visits were for pollen rewards. Therefore the benefit of providing pollen that is accessible to bee foragers outweighs any potential costs to fitness in terms of gender interference in this species.

## Introduction

The foraging behaviour of insects on plants has evolved to optimise reward gain [1], [2]. Such optimisation of foraging can have diverse consequences for the fitness of insect-pollinated plants because their animal visitors often discriminate between individual plants according to reward levels [3], [4]. Among insect-pollinated plants, nectar and pollen are the most common rewards offered to visitors, with plants often offering both simultaneously [5]. Indeed insect pollinators, such as social bees, can often assess potential returns for both nectar [6] and pollen [7], [8] prior to visiting flowers. In addition, both solitary and social bees can vary in their preference for pollen and nectar rewards according to time of day [9]–[11]. Consequently, in hermaphroditic plants with separation of male and female sex functions (dichogamy), pollen-collecting pollinators may neglect female-phase flowers. Such differences in reward preference of pollinators can potentially cause pollen limitation of fecundity [12], [13].

Pollen is an important protein-rich reward for insects, but also functions as the container of male gametes in plants, so consumption by insects can severely reduce fecundity [14]. While this would often be the case for plant male function, the outcomes of such pollen consumption are less clear for plant female function. The most obvious consequence of pollen production is that it can lead to increased seed set due to increased visitation from pollen-collecting bee pollinators [13]. A less obvious consequence is that it can reduce fecundity due to increased interference from self-pollen in plants with late-acting self-sterility [15], [16]. This counterintuitive phenomenon has been demonstrated by means of experimental emasculation [16], [17]. Hence, for self-incompatible hermaphroditic plants, fecundity may be compromised by conflict between male and female sex functions [18], which may be directly influenced by pollinator reward preferences.

In this study we investigate the effect of pollen reward provision on the fecundity of the nectar-producing species *Aloe tenuior*. Most members of *Aloe* are bird-pollinated, where birds visit flowers for the dilute nectar (typically ∼10–15% sugar concentration) [19]. Recently, bee pollination has been recorded in some *Aloe* species [20], [21]. While bees also visit predominately bird-pollinated aloe species, bees tend to be inefficient pollinators compared with birds in these species, with relatively few seeds produced when pollinated by bees [13]. In a previous study on the bird-pollinated *A. maculata*, bees were found to visit emasculated plants less frequently than non-emasculated controls, suggesting that the presence of pollen is a major attractant of bees to aloe flowers [13]. Indeed, Hargreaves and colleagues [18] found that aloes with both strong dichogamy and inaccessible nectar were most prone to pollen theft. Consequently, they suggested on the basis of its relatively short-tubed flowers, that *A. tenuior* is probably bee-pollinated. If so, responses of insects to nectar and pollen rewards and their visitation frequency would determine fecundity in *A. tenuior*. The aims of this study were to establish for *A. tenuior*; (1) whether self-pollen can interfere with seed production, 2) whether foraging preferences of insects vary temporally, 3) if there is a temporal dimension to whether insects actively discriminate between pollen-rewarding and emasculated flowers, resulting in differences in pollen deposition, and 4) whether emasculation would have an overall negative or positive effect on fecundity.

## Materials and Methods

### Study species

**Figure 1:**
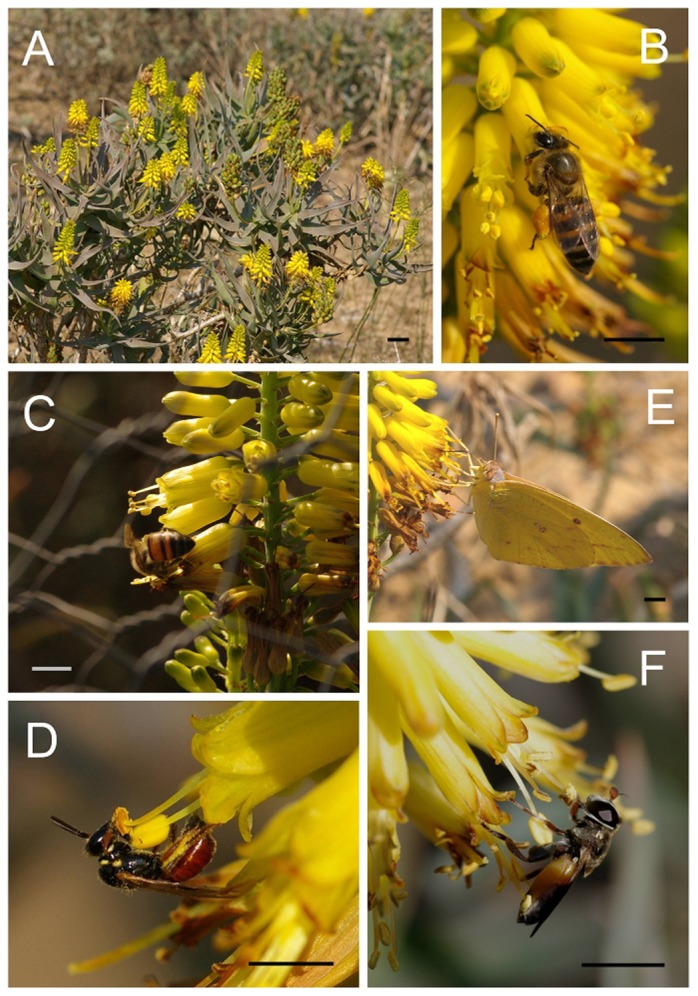
*Aloe tenuior*; (A) plant growth form, (B) native honeybee (*Apis mellifera scutellata*) collecting pollen, (C) native honeybee drinking nectar within bird exclosure cage, (D) solitary bee, *Allodapula* sp. (Anthophorinae) collecting pollen, (E) long-tongued butterfly, *Catopsilia florella* (Pieridae) feeding on nectar, (F) fly feeding on pollen. All scale bars = 5 mm, except A = 5 cm.

*Aloe tenuior* is a relatively small (0.5–1 m), perennial, herbaceous aloe, with approx. 12–20 thin, slightly fleshy leaves and numerous bright yellow flowers borne on multiple inflorescences (Fig. 1). Flowers contain six exserted anthers that are accessible to floral visitors, with nectar at the base of the corolla tube. Inflorescences open acropetally and are protandrous, with male-phase lasting 48 hr (K.J. Duffy pers. obs.). We studied a large population (at least 2500 flowering individuals) of *A. tenuior* near the Great Fish River (S33° 06' 49'', E26° 55' 52'') in the Eastern Cape, South Africa between July – August 2010, May – June 2011, and May 2013. At this site in winter *A. tenuior* was the dominant flowering plant, with few other plants flowering, and no others mass flowering to the same extent as *A. tenuior* and regularly visited by bees (K.J. Duffy pers. obs.). *Aloe tenuior* is a species of least concern in South Africa and is not threatened (http://redlist.sanbi.org/species.php?species=2206-252; accessed 30^th^ August 2013). Permission to access to the study population was not required as the study population occurs in communal land in the Eastern Cape. Permission to collect plant material was granted under permit number CRO 160/11CR.

### Breeding system and pollen tube growth

We determined the breeding system of *A. tenuior* in 2010 by performing hand pollination of flowers with either cross- or self-pollen. Ten inflorescences were bagged prior to anthesis using 1 mm aperture nylon bags supported over inflorescences with a wire cage. We added either self- or cross-pollen separately to ten flowers on one inflorescence of each of five separate plants, with excess flowers removed (so that self-pollen would not be transferred on the nylon bags). Differences between the two treatments were analysed using Generalised Estimating Equations (GEEs) [22] with a Poisson error structure using the geepack package [23] in R [24]. For this and subsequent GEE analyses, we used individual plant as a grouping variable to account for repeated measures on individuals and an exchangeable correlation matrix. As GEE analysis cannot be conducted when one treatment has only zeros, we artificially added one seed to the self-treatment to generate the model.

To establish the location of a potential self-sterility barrier in *A. tenuior*, in May 2013 we hand-pollinated individual stigmas of seven *A. tenuior* plants with either self- or cross-pollen and repeated this treatment 24 hours later to ensure stigma receptivity. Twenty-four hours after the second addition of pollen, pistils were softened in 2 M NaOH for 18 h, rinsed in distilled water for 1 h and stained in a 0.1% solution of aniline blue in 0.1 M K_2_HPO_4_ for 4 h. Pistils were mounted in a drop of stain on a microscope slide and squashed under a coverslip. Pollen tube growth was examined in the stigmas and ovules. For each treatment we counted all visible ovules and checked for the presence of pollen tubes. Differences between the two treatments in the proportion of ovules that were penetrated by pollen were analysed with a generalized linear model [25] with a binomial error structure in R.

### Floral visitors and nectar rewards

We tested whether *A. tenuior* depends on insect pollinators by placing 17 potted plants in a shaded greenhouse at the Rhodes University botanic garden. This environment contained no pollinators, so we could examine whether *A. tenuior* depends on pollinators. As members of *Aloe* are commonly bird-pollinated [18], in 2010 we performed a selective exclusion experiment involving 20 mm aperture wire cages to establish whether birds make a contribution to seed set of *A. tenuior*. These cages allowed insects to visit flowers (Fig. 1c), but excluded birds [26]. We tested for differences in seed production between caged individuals and controls using GEEs with plant as a grouping factor, an exchangeable correlation matrix, and a Poisson error distribution. In 2010 and 2011 we captured and identified representatives of all visitor taxa and recorded their pollen loads. We quantified pollen loads carried by visitor taxa by removing pollen from the insect with fuschin gel and transferring them to a microscope slide. The number of pollen grains carried per insect was counted using a 20× microscope. As there were large differences in the number of pollen grains on each insect, we categorised the quantity on each insect as <100 grains, 500–1000 grains, 2000–5000 grains, and 7500–10000 grains. Differences in the number of pollen grains carried between visitor groups were analysed at the family level with a Kruskal-Wallis test applied to the ranked categories. A post hoc test was used to analyse significant differences between the pollen loads carried by different insect families with the pgirmess package for R [27]. To test whether floral visitors actively depleted nectar during the day, we quantified nectar volume and concentration from each of three flowers from six bagged and six unbagged individuals in the field between 1100–1300. Nectar volume was measured in the field with 1 µl glass microcapillary tubes. Differences in nectar volume between bagged and unbagged flowers were examined with a one-factor ANOVA, with nectar volume log-transformed prior to analysis. Nectar sugar concentration could not be estimated for unbagged flowers due to small quantities, therefore we estimated nectar sugar concentration from bagged individuals using a handheld refractometer.

### Temporal patterns in reward preference and pollination

On one representative day (13^th^ May 2011) we recorded whether flower visitors partitioned floral resources over the course of a day. Two observers (K.J.D. and C.I.P) made replicated 30 min visitor observation intervals throughout the day in the population resulting in a total of eight observation periods per observer. A different patch was selected for each observation period and we recorded whether visitors foraged for pollen or nectar. This was easily determined in the field, as bees that were foraging for pollen did not enter flowers and collected pollen from the anthers, while nectar-consuming bees entered the corolla tube to collect the nectar from the base of the tube (Figs 1c,d) [28]. To determine temporal patterns we plotted a time-series graph of pollen and nectar visits. We assessed temporal autocorrelation in reward usage using partial autocorrelation plots in R by converting the time of each observation period into a time-series object. This allows us to describe the relationship in reward preference in each time period, while controlling for the correlations between all successive time periods. We also fitted curves to the relationships between time of day and the total number of bees visiting *A. tenuior* using both linear and quadratic regression. We tested for the effect of time of day on the proportion of pollen visits among the total number of visits observed using quadratic regression.

To test whether diurnal variation in reward preference of visitors has an impact on pollination success, we quantified pollen deposition on stigmas on one representative day (22^nd^ May 2011). Forty plants were selected with twenty plants assigned to an emasculation treatment (pollen rewards absent) and twenty assigned as an unmanipulated control. On each plant, a total of twenty flowers (ten flowers on two inflorescences) were either emasculated or left unmanipulated with all excess flowers removed. Ten plant pairs were assigned to a ‘morning’ exposure to visitors (1030–1230) and ten plant pairs were assigned to ‘afternoon’ exposure (1330–1530). Inflorescences were bagged at the end of each exposure period and removed from the field. To visualise pollen grains on stigmas, stigmas were removed and mounted on a glass slide with fuschin gel. Pollen grains deposited on stigmas were counted under a 40× compound microscope. Due to non-independence of repeated pollen counts on the same plant we used GEEs [23] with a Poisson error distribution. Time of day (morning or afternoon) and treatment (emasculated or non-emasculated) were explanatory variables. To test whether pollen deposition patterns reflected visitor preferences, we made 2×15-minute observations of visitor foraging on emasculated and non-emasculated plants in the same morning and afternoon periods. We analysed these data (counts of visitors) with a generalised linear model (GLM) [25] corrected for overdispersion with a Poisson error distribution and a log link.

### Overall effect of emasculation on fecundity

To test for the overall effect of pollen provision on flower visitor attraction and plant fecundity, in 2010 we made replicate 30-minute observation periods of flower visitation rates over four days to both emasculated and non-emasculated plants (n = 8 for both treatments). Prior to observations, all open flowers on one plant were emasculated and the insect visitation rate was compared with a non-emasculated control within 2 metres. Plants were matched for the number of open flowers to control for floral display size, as this may affect attraction of bees to plants. Observations were made randomly between 1030 and 1530. To quantify the effect of the presence of self-pollen on fecundity, in 2011 thirty pairs of *A. tenuior* were selected at random. On each plant, two inflorescences were either subjected to an emasculation treatment or as an unmanipulated control. To control for floral display size, all but ten flowers were removed on each of two inflorescences, leaving 20 flowers on each plant. At the end of the flowering season we recorded the number of fruit set, and mean number of seed set per fruit. Differences in insect visitation rate, fruit set, seeds per fruit, and aborted seeds per fruit between emasculated and non-emasculated plants were analysed with separate generalised linear models [25] with either a binomial or Poisson error structure corrected for overdispersion in R.

## Results

### Breeding system and pollen tubes

**Figure 2:**
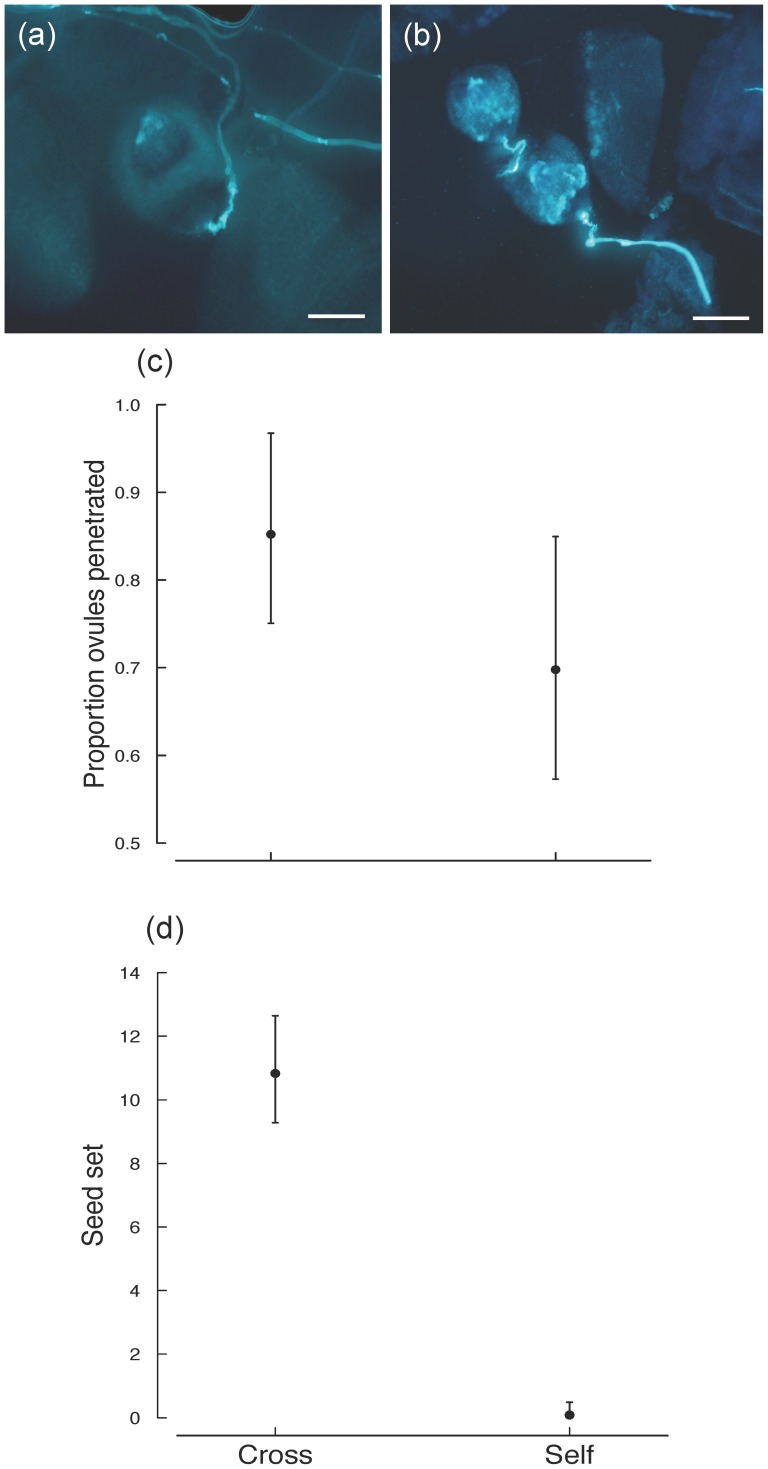
Florescence micrographs showing pollen tubes associated with individual ovules and corresponding effects on ovule penetration and seed set in *Aloe tenuior*. Ovules are penetrated by both: (a) Cross-pollen tubes; and (b) self-pollen tubes (scale bars = 100 µm). The mean proportion of ovules penetrated (c) does not differ between cross- and self-pollinated flowers, but seed set (d) is significantly greater for cross-pollinated flowers. Means (±SE) are back-transformed.

No fruits were produced when only self-pollen was added to flowers, indicating the presence of a self-incompatibility system in *A. tenuior*. We found that both self- and cross-pollen tubes penetrate the micropyle in the ovules of *A. tenuior* (Fig. 2a, b). There were no differences between the proportion of ovules penetrated by self- and cross-pollen (Z = 1.54; df = 13; P = 0.125; Fig. 2c). However, there were strong differences between selfed and crossed treatments, with seed set substantially higher in crossed individuals than in selfed individuals (Wald χ^2^ = 32.04; P<0.001; Fig. 2d).

### Floral visitors and nectar rewards

Plants that were removed from the field and put in a pollinator-excluded greenhouse failed to set any fruit, indicating that they require a pollinator to set seed. Plants from which birds were excluded produced similar numbers of seeds per fruit to uncaged plants, indicating that insects are the primary pollinators of *A. tenuior* (Wald χ^2^ = 2.42; P = 0.12). We observed birds present at the study population, but none were observed visiting flowers of *A. tenuior*. A total of 19 insect taxa visited *A. tenuior* (Table 1). Members of the bee families Apidae and Halictidae and members of the butterfly families Hesperiidae, Lycaenidae Nymphalidae, and Pieridae were found visiting *A. tenuior* flowers. There were significant differences between visitor taxa in the size of their pollen loads (H_5_ = 26.46; P<0.001) with members of the Apidae and Halictidae carrying more pollen than members of the Hesperiidae, Lycaenidae Nymphalidae, and Pieridae (P<0.05), indicating that bee visitors carry more *A. tenuior* pollen compared with butterfly visitors. There was a significant difference in nectar volume between bagged and unbagged plants (F_1,12_ = 8.719; P = 0.012) with bagged plants containing more nectar (mean = 1.06 µl ±S.E. 0.17) than unbagged plants (mean = 0.38 µl ±S.E. 0.09), indicating that floral visitors depleted nectar during the day. Mean nectar sugar concentration in bagged plants was 34.3% (±S.E. 2.8%).

**Table 1:** List of taxa/insect morphotypes collected visiting flowers of *A. tenuior* and median pollen loads carried.

Taxon/Morphotype	Family	N	Median pollen load carried
*Apis mellifera*	Apidae	6	7500
Large *Allodapula* sp.	Apidae	2	625
Medium *Allodapula* sp.	Apidae	4	750
Small *Allodapula* sp.	Apidae	2	517.5
Halictid sp.	Halictidae	2	5000
*Chrysoritis chrysaor*	Lycaenidae	3	0
*Junonia hierta cebrene*	Nymphalidae	1	19
*Precis archesia archesia*	Nymphalidae	2	17.5
*Vanessa cardui*	Nymphalidae	1	16
*Belanois gidica abyssinica*	Pieridae	6	13
*Catopsilia florella*	Pieridae	2	3
*Colotis eris eris*	Pieridae	2	17.5
*Colotis evagore antigore*	Pieridae	1	0
*Dixeia charina charina*	Pieridae	1	0
*Nepheronia buquetii*	Pieridae	1	70
*Pontia helice*	Pieridae	2	0
*Pinacopteryx eriphia*	Pieridae	1	17

N = number of individuals.

### Temporal patterns in reward preference and pollination

**Figure 3:**
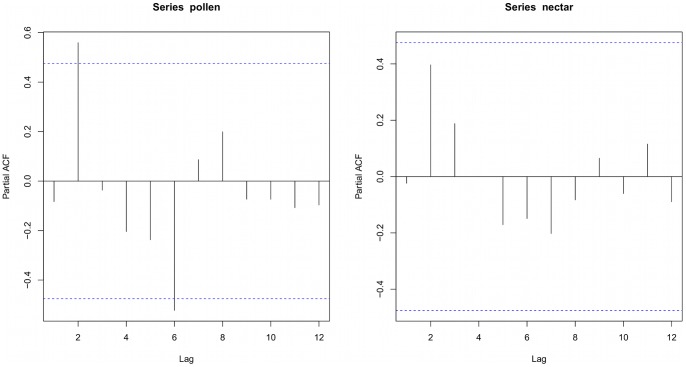
Partial autocorrelation plots of bee visit preference for pollen and nectar. Each observation period is denoted by a lag. The dashed blue line is the threshold to show where there is significant partial autocorrelation in each lag.

**Figure 4:**
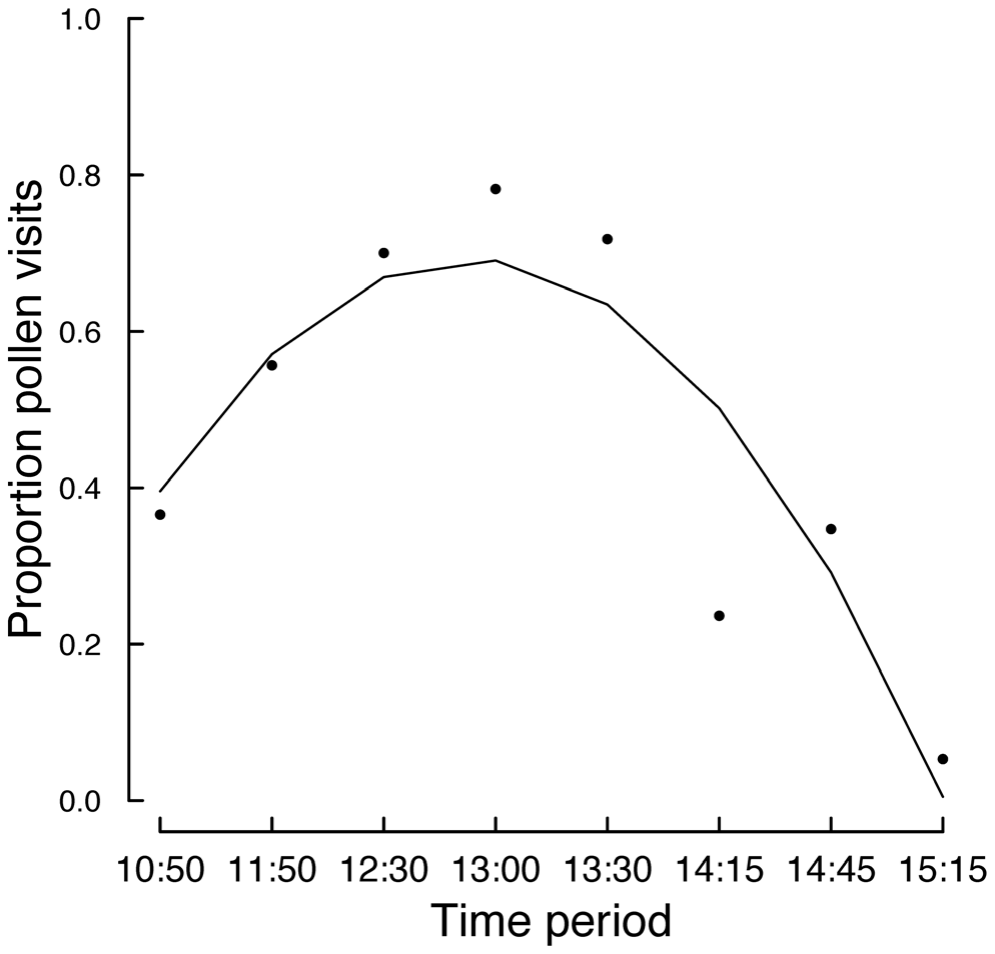
Temporal patterns in floral reward preference; closed circles, the proportion of pollen visits. Fitted line is based on quadratic regression. Equation of the line: y = 0.856+0.291−0.039x^2^; F_2,5_ = 10.19; P = 0.015; R^2^ = 0.724.

Among bee visitors, native honeybees (*Apis mellifera scutellata*) were the most common (85.4% visits) with solitary bees (halictid and small allodapine bees) less frequent visitors (14.6% of visits). Partial autocorrelation plots revealed some autocorrelation at lag 2 and lag 6 for pollen visits, but no autocorrelation for nectar visits (Fig. 3). Therefore, we retained all observations for our analysis. The number of bees observed visiting *A. tenuior* at each time period did not vary over the course of the day (quadratic regression; F_2,5_ = 0.835, P = 0.24). However, there was a clear temporal pattern in pollinator visitation according to reward use, with pollen visits increasing during the day then decreasing later in the day, with the opposite pattern observed for nectar visits (Fig. 4).

**Figure 5:**
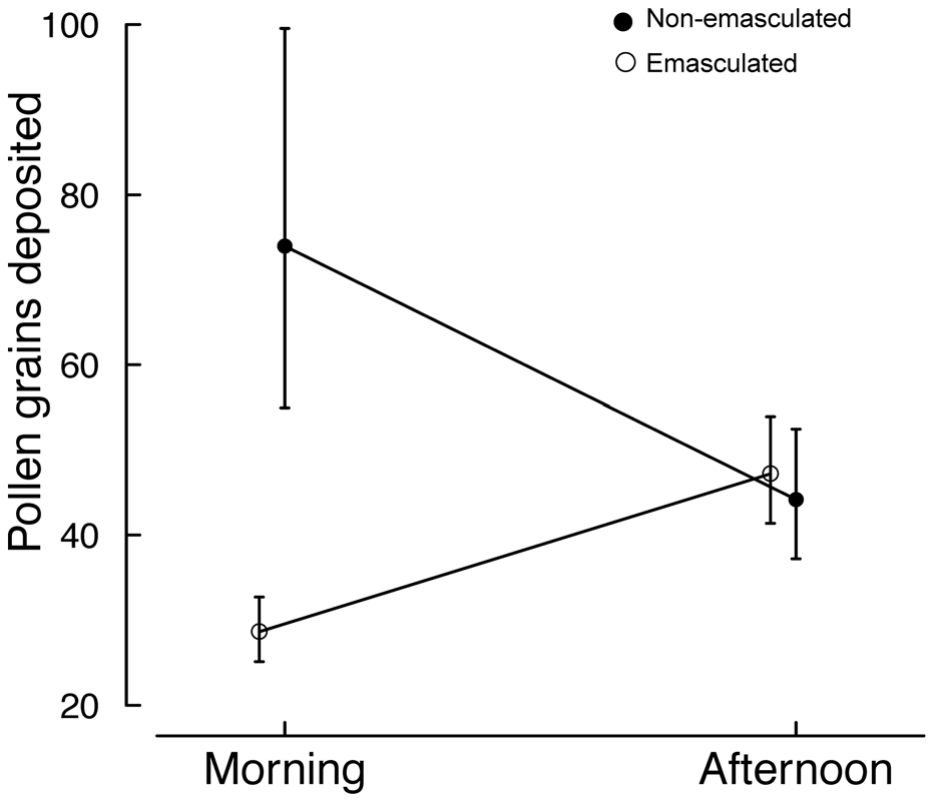
Effect of emasculation on the mean number of pollen grains deposited per stigma on emasculated (open circles) and intact (closed circles) individuals in the morning (exposure to pollen-collecting bees) and afternoon (exposure to nectar-collecting bees). Means (±SE) are back-transformed.

The number of pollen grains deposited on stigmas of *A. tenuior* varied according to treatments and time periods (Wald χ^2^ = 6.37; P = 0.013; Fig. 5). Pollen deposition on intact (non-emasculated) flowers declined in the afternoon and emasculation greatly reduced pollen deposition in the morning but had little effect in the afternoon. Since emasculated flowers cannot self-pollinate, we infer that most of the pollen deposited on flowers in the afternoon was cross-pollen. This was reflected in visitation rate analysis, where we found that there was a significant difference in both the number of visits to emasculated plants compared with intact plants in the morning, and a significant decrease in the number of visits to intact plants in the afternoon compared with the morning (t = 4.302; P = 0.013).

### Overall effect of emasculation on fecundity

**Figure 6:**
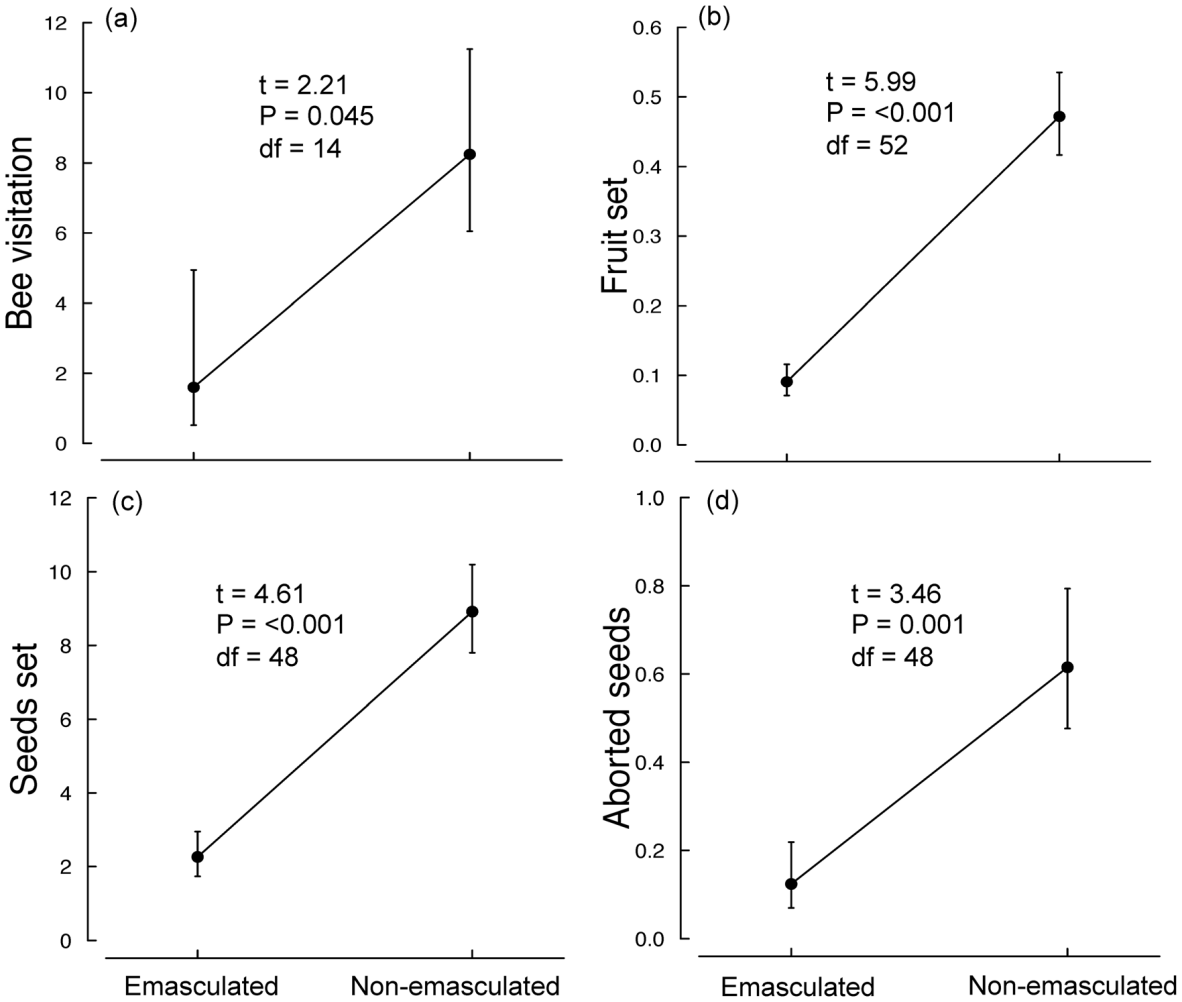
The effect of emasculation of *A. tenuior* flowers on: (a) bee visitation rate per 30 mins per plant, (b) proportion fruit set, (c) mean seed set per fruit, and (d) mean number of aborted seeds per fruit. Means (±SE) are back-transformed.

Plants with flowers that were emasculated throughout anthesis exhibited reduced visitation rates (Fig. 6a), fruit set (Fig. 6b), seeds per fruit (Fig. 6c), and aborted seeds per fruit (Fig. 6d).

## Discussion

We found that native honeybees and solitary bees are the primary pollinators of *A. tenuior* and are attracted mainly by the presence of exposed pollen in flowers during the day. Emasculation resulted in reduced fecundity in *A. tenuior* individuals (Fig. 6). This is similar to a previous study on *Aloe maculata* showing that when pollen was removed from flowers there was an overall decrease in bee visitation and fecundity [13]. However, as found in *A. maculata*, emasculation also reduced seed abortion (Fig 6d), which can be attributed to reduction in gender conflict arising when self-pollen disables ovules.

A novel aspect of this study is that it shows how different patterns in reward use by bee pollinators can impact plant fecundity. Quantifying the behavioural and ecological determinants of bee activity has been of interest to researchers for many years, yet how such changes in bee activity affects plant fecundity is still poorly understood [2]. We observed strong temporal patterns in pollinator reward preference, which appear to have contrasting implications for pollen deposition. The switch to nectar foraging in the late afternoon is reflected in reduced visitation to pollen-containing flowers at this time (Fig. 4) and pollen deposition on stigmas of intact flowers in the afternoon compared with the morning (Fig. 5). Similar temporal partitioning of floral reward use by bees has been found in earlier studies. For example, Kay and colleagues [29] reported that foraging bumblebees and honeybees could distinguish between male and female flowers of the dioecious *Silene dioica*, and that bumblebees switched from male to female flowers in the afternoon. Indeed, Stone and colleagues [11] showed that there was a bimodal pattern in foraging for pollen and nectar in the solitary bee *Anthophora pauperata* and that this was driven by relative availability of pollen and nectar from its main food source, the protandrous hermaphrodite plant *Alkanna orientalis*. However, the direction of the temporal pattern of reward use by pollinators (i.e. whether they forage for nectar before pollen or vice versa) should not be expected to influence the net outcome on plant fecundity.

Although we found a relative increase in pollen collection in the middle of the day, bees make occasional visits for nectar to sustain their foraging during this time period. The concentration of nectar (∼35% w/w) is in the range of sugar concentration for bee-pollinated aloes [20]–[21] and there were higher nectar volumes in bagged plants, indicating nectar consumption by bees during the day. Emasculation had little effect on pollen deposition in the afternoon, suggesting firstly that bees were not responding to pollen rewards at that time and secondly that the bulk of pollen deposited on stigmas, even of intact flowers, at this time is cross pollen. Although they experience a reduced quantity of pollen deposited, plants pollinated in the afternoon would have increased quality of seeds as there would be less gender conflict arising from potential ovule discounting [13], [15]–[16], [30]. It may be that although we found there are temporal patterns in pollinator reward preference, the energy requirements of individual bees may occasionally obscure this relationship on an individual basis. Indeed, Herrera (1990) showed that the daily activity patterns of insect pollinators did not directly match the daily floral cycle of *Lavandula latifolia*, which provides both nectar and pollen rewards [31]. However, in a later study Herrera (2000) showed that *L. latifolia* flowers that opened in the daytime (0930–1630) had higher fecundity than those that flowered at dawn or dusk, which was probably due to great activity of bee and butterfly pollinators during the daytime [32]. Therefore, *A. tenuior* probably benefits in offering both pollen and nectar rewards, with pollen rewards increasing visitation rates during the morning, while offering a nectar reward makes the flowers energetically attractive in the afternoon and, importantly, ensures that cross-pollen is deposited on female-phase flowers.

The mechanism underlying the ability of bees to distinguish between different floral rewards in plants needs to be investigated further. In plants that provide pollen and nectar rewards, the trade-off between pollen and nectar production can be related to bee visitation rates. For instance, Thomson and colleagues (1989) [6] showed that bumblebees discover individual plants of *Aralia hispida* with elevated levels of either nectar or pollen and return to more rewarding plants more frequently. They found that pollen production increases towards the end of flowering, which resulted in more efficient pollen transfer than simultaneous presentation of rewards [6]. Our artificial removal of anthers from flowers presumably removes the visual cue of pollen, however it has yet to be established if exposed anthers are a strong visual cue for a pollen reward for bees. It could be that the removal of pollen removes the odour of pollenkitt, which is known to be a chemical scent cue to solitary bees [33]–[35]. Regardless, artificial emasculation may remove a short-distance visual cue and when honeybees near flowers they can distinguish between pollen and nectar rewarding individuals. As such foraging decisions can have direct consequences for the fecundity of insect-pollinated plants, this behaviour needs to be further understood in order to better predict how bees respond to plant resource availability and how it affects the long-term fitness of bee-pollinated plants.

In conclusion, this study demonstrates that there is a strong temporal dimension to the effects that nectar and pollen rewards can have on fecundity of self-incompatible bee-pollinated plants. Our results suggest that providing a pollen reward to bees along with nectar represents an optimal strategy to ensure fecundity in *A. tenuior*.
